# Same-Day Discharge after Laparoscopic Appendectomy for Simple Appendicitis in Pediatric Patients—Is It Possible?

**DOI:** 10.3390/children9081220

**Published:** 2022-08-12

**Authors:** Miro Jukić, Alexander Tesch, Jakov Todorić, Tomislav Šušnjar, Klaudio Pjer Milunović, Tomislav Barić, Zenon Pogorelić

**Affiliations:** 1 Department of Pediatric Surgery, University Hospital of Split, Spinčićeva 1, 21 000 Split, Croatia; 2 Department of Surgery, School of Medicine, University of Split, Šoltanska 2, 21 000 Split, Croatia

**Keywords:** acute appendicitis, one-day discharge, complications, pediatric surgery, children, laparoscopic appendectomy, same-day discharge

## Abstract

(1) Background: One-day surgery has been widely adopted for many elective laparoscopic procedures in pediatric patients. Recently, the same protocol has been investigated for some emergency procedures, such as laparoscopic appendectomy. This study aimed to evaluate the safety and effectiveness of discharge from hospital within 24 h in pediatric patients who received laparoscopic appendectomy for uncomplicated acute appendicitis. (2) Methods: From 1 March 2021 to 1 May 2022, a total of 180 pediatric patients who were discharged from hospital within 24 h after laparoscopic appendectomy for uncomplicated appendicitis were included in this prospective single-center study. The primary outcome of this study was the safety of discharge from hospital within 24 h after laparoscopic appendectomy for uncomplicated appendicitis, as well as the parental satisfaction with this protocol. Secondary outcomes included the rate of readmission or unplanned return to the operating room, the complication rate and a cost-effectiveness analysis. For each patient, demographic data, preoperative evaluation (physical examination, laboratory data, imaging), duration of surgery, intraoperative and postoperative complications, length of hospital stay and pain levels, as well as parental satisfaction with this protocol, were recorded. (3) Results: The median age was 11 years (interquartile range (IQR) 10, 14). The majority of the patients (63.8%) were males. The median length of hospital stay after surgery was 15 h (IQR 12, 19). The highest level of satisfaction, at discharge, was recorded in most of the respondents (*n* = 155, 86.1%), while the remaining 25 (13.9%) expressed moderate levels of satisfaction. The median pain levels according to a visual analogue scale for all postoperative days were low (range 0–4). In four patients (2.2%), unplanned readmission before the seventh postoperative day because of postoperative intraabdominal abscess was recorded. All patients with abscess formation were treated conservatively. The majority of the parents (*n* = 175; 97.2%) expressed the highest level of satisfaction during the outpatient follow-up examination on the seventh postoperative day. (4) Conclusions: Same-day discharge after laparoscopic appendectomy for simple appendicitis in pediatric patients was safe and feasible. Parental satisfaction with this protocol was very high. With the right protocol and parent education, pediatric patients who underwent laparoscopic appendectomy because of non-complicated acute appendicitis may be successfully treated in this way.

## 1. Introduction

Acute appendicitis (AA) is one of the most common causes of abdominal pain in children, and as such, it belongs to the high-volume disease entities requiring hospitalization. In fact, it is the most common surgical emergency in pediatric patients [1]. Compared to adults, in pediatric patients, there is an increased risk for complicated AA, which leads to a prolonged hospital stay. The incidence of appendicitis, in the pediatric population, is estimated to be the highest between the ages of 12 and 17 years [2]. The lifetime risk of AA is estimated to be 8.6% in males and 6.7% in females, with a lifetime probability of appendectomy of 12% for males and 23% for females [3,4].

While rural areas report a slightly lower incidence, the data from newly industrialized countries suggest a rapid rise in the incidence of AA. The incidence of uncomplicated AA is progressively declining worldwide, while the incidence of complicated AA seems more constant in numbers [5]. In children, if the diagnosis of AA is undetected and untreated, it leads to perforation in 35–47% of cases, with the highest rates in children under the age of five [6].

Patients may present themselves at different stages of AA. More precisely speaking, AA encompasses a range of diseases that are called uncomplicated in its earliest stages and complicated AA in later stages, which are often characterized by perforation or abscess. During early uncomplicated AA, an appendix may present macroscopically without inflammatory signs. In general, complicated AA warrants longer hospital observation and treatment [7]. Gomes et al. proposed an intraoperative scoring system grading appendicitis into five groups. Table 1 outlines the comprehensive staging according to intraoperative findings [7].

During the late 20th century, implementing the laparoscopic technique in the operating room to treat AA heralded a new era of approaching the disease [8]. Since then, hospitalization after laparoscopic appendectomies shortened to only two days [9,10]. Over the past years a variety of surgeries, including laparoscopic interventions, have been performed in an outpatient manner, which could be translated to laparoscopic appendectomies [11]. Against this background and due to good experience with fast-track surgery protocols, the idea of same-day discharge (SDD) after laparoscopic appendectomy for AA in children was born [11,12]. In this study, SDD or one-day discharge is defined as discharge from hospital within 24 h after laparoscopic surgery.

One-day discharge protocols for laparoscopic appendectomy have been evaluated since the start of the 2010s; yet, the exact definition may differ. The implementation of such protocols affects all levels of hospital care and demands a well communicated pathway from admission to the release of the patient. Once applied, SDD may minimize inpatient resources for this high-volume disease entity, decrease nosocomial infections and show positive outcomes for patient and family satisfaction [12].

Stress in children due to hospitalization has been thoroughly investigated. Postoperative behavioral changes, including separation anxiety, tantrums, fear of strangers, eating problems, nightmares, night terrors and enuresis, have been observed. These changes may last as long as a full year. Young age, prior negative experience with hospitals or medical care, hospitalization, postoperative pain, parental anxiety and certain personality traits of the child make up major risk factors for postoperative behavior problems [13]. The shortening of visiting hours and a stricter policy for visitors in hospitals around the world due to the COVID-19 pandemic may further enhance these effects.

Parental anxiety has an effect on the distress levels of the hospitalized child, which may continue into the post hospitalization period and, on occasion, may even increase postoperative recovery time [14]. It has been described that parents as well as their children report not feeling educationally and emotionally well prepared for an outpatient surgical setting [15]. Parental counseling should be thoroughly performed, as this is a new approach and parents may be misled by other healthcare professionals about the safety of this protocol [12]. Standardizing instructions via trained staff members improves parent understanding [16]. Child-life specialists may play a key role here in preparing families for the surgical experience. This way, parents may be reassured about the treatment strategy, and they may be equipped with knowledge to support their child through the surgical and recovery process [17]. For same-day-discharge laparoscopic appendectomy, Aguyao et al. described three phases of consulting with parents: the first time, the moment a diagnosis of AA is on hand; the second time, in the preoperative holding area; and the last time, immediately postoperatively by the attending surgeon [18]. For home management, pain control using paracetamol alternating with scheduled ibuprofen was successfully shown in two studies investigating parental satisfaction [19,20]. Ngo et al. additionally pointed out the need to educate parents about possible postoperative constipation of the child and how to prevent it [20].

The aim of this study was to evaluate the safety and effectiveness of discharge from hospital within 24 h in pediatric patients who received laparoscopic appendectomy for uncomplicated AA. Additionally, parental satisfaction was investigated for this protocol.

## 2. Materials and Methods

### 2.1. Patients

This prospective cohort study included 180 children with a median age of 11 (interquartile range (IQR) 10, 14) years who underwent surgery at Department of Pediatric Surgery, University Hospital of Split, because of uncomplicated AA, in the period from 1 March 2021 to 1 May 2022. Patients of both genders, between 6 and 17 years of age, who underwent laparoscopic appendectomy for uncomplicated AA and were discharged within 24 h after surgery were included in the study. Patients out of the predetermined age range, those operated on due to complicated AA, patients who had a duration of hospital stay >24 h after surgery and patients who underwent an open operating technique for AA were excluded from the study. The study was approved by the ethics review board of our hospital (reference number 500-03/21-01/151; date of approval: 28 February 2021).

### 2.2. Outcomes of the Study

The primary outcome of this study was the safety of discharge from hospital within 24 h after laparoscopic appendectomy for uncomplicated appendicitis, as well as the parental satisfaction with this protocol. Secondary outcomes included the rate of readmission or unplanned return to the operating room, the complication rate and a cost-effectiveness analysis.

### 2.3. Study Design

Patients presenting with AA to the surgical emergency department went through the diagnostic algorithm (clinical examination, acute inflammatory laboratory markers, abdominal ultrasound) and were evaluated using the Appendicitis Inflammatory Response (AIR) score [21]. The intraoperative decision of inclusion was made by the operating surgeon, and the parents were informed about the possibility of participation in this study. Before discharge the parents were counseled about pain management and home child care. The two-page questionnaire was handed to the parents with instructions for use. The questionnaire was designed to investigate possible changes in behavior and the pain level of the child using a visual analogue scale (VAS) measuring from 0 to 10. With this questionnaire, home-based pain management was recorded. Parents returned to the outpatient check-up on the seventh postoperative day and handed in the filled-out questionnaires. Complication was defined as any postsurgical condition requiring emergency visit, readmission or return to the operating room. Patients were evaluated for surgically related complications using the Clavien–Dindo classification [22].

### 2.4. Surgical Technique

The standard of procedure at our Department comprises a laparoscopic 3-port approach. Depending on the age and weight of the young patient, a pneumoperitoneum using CO_2_ at 6–12 mmHg is established. A 5 mm trocar is placed supraumbilicaly, through which a laparoscope (Olympus, Tokyo, Japan) is inserted, and a first inspection of the abdominal cavity is performed. Another 5 mm trocar is placed under the right costal arch, and a third working 10 mm trocar is placed either in the left lower quadrant or suprapubically. The preparation of the appendix is performed using a longitudinal (Ultracision™; Ethicon Endo-surgery, Cincinnati, OH, USA) or torsional harmonic scalpel (Lotus™; BOWA-electronic GmbH, Gomaringen, Germany) [23]. The appendiceal base is secured using a polymeric clip (Ligating Clips XL; Grena, Brentford, UK) [24] or repeated applications of a harmonic scalpel in a stepwise manner [25]. In harmonic-scalpel appendectomy, the appendiceal base is secured by repeatedly using the harmonic scalpel to obliterate the lumen of the appendix. The specimen is then retrieved through the 10 mm trocar in an endoscopic bag (Ecosac EMP 70; Espiner Medical Ltd.; Measham; UK). The abdominal cavity is rinsed with normal warm saline solution, and the stump is checked for leakage before retracting all trocars and closing the skin with a interrupted nylon closing suture (Surgipro™ II; polypropylene 3/0; Covidien, Dublin, Ireland).

### 2.5. Postoperative Protocol and Follow-Up

After surgical procedure, the patients were observed in the intensive care unit for several hours until they were fully awake. An intravenous fluid (5% glucose + 0.45% NaCl) in a dose depending on the child’s weight and daily requirements was started immediately postoperatively. Peroral-fluid intake was started two hours after surgery. If the patient tolerated the fluid well, a dietary postoperative meal was started two hours after fluid intake without nausea. Paracetamol (Perfalgan; Bristol-Myers Squibb Pharmaceuticals limited, Bristol, UK) at a dose of 10–15 mg/kg was used for analgesia. Afebrile patients with no vomiting within 12–20 h and complete tolerance of oral meals were discharged from the hospital depending on the decision of the operating surgeon. The children underwent a follow-up on the seventh day after discharge at our outpatient clinic.

None of the patients received antibiotics perioperatively, preoperatively, intraoperatively nor postoperatively, except for the four patients who had unplanned readmission and were treated with an antibiotic combination (Gentamicin and Metronidazole) for 5–8 days depending on clinical presentation, US reduction in the abscess and improvement of laboratory results.

### 2.6. Statistical Analysis

SPSS 24.0 software (IBM Corp., Armonk, NY, USA) was used for the analysis of the data. The medians and interquartile ranges (IQRs) were used to express the distribution of the quantitative data, while absolute numbers and percentages were used to describe the categorical data.

## 3. Results

The demographic data of the patients are summarized in Table 2. A total of 90 patients (75.6%) had positive findings upon US examination. False-negative ultrasound findings were observed in 29 patients (24.4%).

### 3.1. Outcomes of Treatment

The intraoperative finding was positive for AA in the majority of the cases (*n* = 164, 91.1%). Among the 164 patients with positive appendicitis, the most common finding at pathohistology was the phlegmonous type of AA (*n* = 110, 67%), followed by the gangrenous type (*n* = 50, 30.5%). Four cases of initially inflamed catarrhal appendices (2.5%) were identified. The harmonic scalpel was most commonly used for securing the appendiceal base (*n* = 155, 86.1%), while for the rest, a polymeric clip was used (*n* = 25, 13.9%). The median of operative time was 20 min (IQR 16, 25). The median length of the postoperative stay (LOS) was 15 h (IQR 12, 19). The treatment-outcome data are summarized in Table 3.

### 3.2. Complications

There were no immediate intraoperative complications registered. Four patients (2.2%) had an unplanned readmission prior to the seventh postoperative day. According to the Clavien–Dindo classification, all four patients were graded as grade II and were treated conservatively with antibiotics due to the minimal formation of abscess (Table 4). None of the patients had an unplanned return to the operating room. No other emergency visits were registered.

### 3.3. Parental Satisfaction

A total of 155 parents (86.1%) graded the highest satisfaction score at the time of discharge, while 25 parents (13.9%) reported mediocre satisfaction (Table 5). Out of the 25 parents with mediocre scores on the day of outpatient appointment, 20 parents (80%) changed to the highest satisfaction, and only 5 parents (20%) kept the mediocre score.

**Figure 1:**
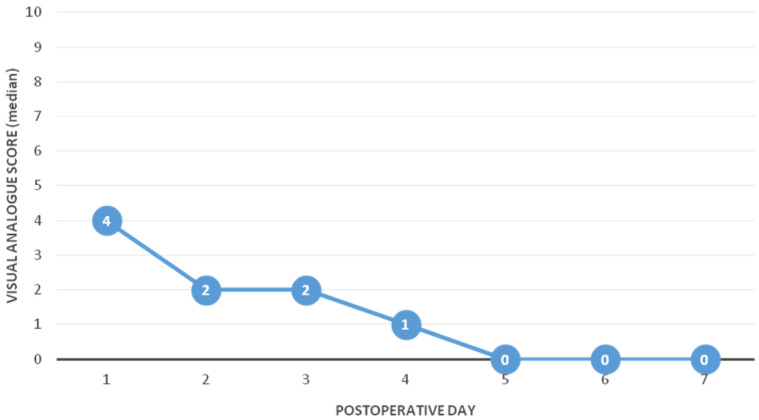
Postoperative pain assessment. Data are presented as medians ± interquartile ranges.

The medians of the pain level according to the VAS for all postoperative days were low: 4 (IQR 4, 6), 2 (IQR 2, 5), 2 (IQR 0, 4), 1 (IQR 0, 2), 0 (IQR 0, 2) and 0 (IQR 0, 0) for the first, second, third, fourth, fifth, and sixth and seventh postoperative days, respectively (Figure 1). In the majority of the cases, for pain control, paracetamol was used on the first postoperative day.

## 4. Discussion

Acute appendicitis is one of the leading causes of emergency surgery in the pediatric population [1]. While there is still an ongoing debate about the gold-standard therapeutic approach, the outcomes of laparoscopic operative treatment have been shown to have superior results. Children recover and are discharged after a short hospital stay [26]. Hospitalization is coupled with the separation from the parents, which may add additional stress not only to the child but the whole family [14,17]. During the COVID-19 pandemic, hospital visits were even more limited, which led to the possible unnecessary separation of children from their parents at a young age [27]. Already before the COVID-19 pandemic, treatment protocols for laparoscopic appendectomies for uncomplicated AA and same-day discharge were implemented, showing promising results regarding safety and cost efficiency [19].

The sample size of this study and median age of patients are comparable to existing prospective literature [12,19,28]. Of foremost importance when implementing a new treatment protocol is to rule out any possible new adverse effects for the patient compared with the established standard of procedure. In this study, only four patients, or 2.2%, had an unplanned readmission to the hospital within the first seven postoperative days. All four patients in our study were treated for the minimal formation of periappendicular abscess with antibiotics. A previous evaluation over a three-year period for the quality of surgery at Department of Pediatric Surgery at our institution found unplanned returns to the operating room of 0.47% and readmission rates below 1%. These results include elective surgeries, and the authors found the rates of unplanned return to the operating room for emergency operations to be 4.5 times higher and the readmissions for emergency cases to be 3.2 times higher. The most common reason was appendectomy [29,30]. This result is consistent with previous studies showing readmission rates of up to 2.5% for discharged patient groups [12,18,31]. Cairo et al. reported, in a large retrospective cohort study, readmission for one-day discharge treatment protocols to be up to 1.89%. They also found that there were no differences in the reason for readmission compared to the conventional hospitalization group [32]. Most recently, in 2022, Lo et al. indicated no differences in readmission or emergency visits in a 30-day postoperative period after implementing an early discharge protocol for appendectomy in children [33]. Compared with the literature, the results of this study show safety in implementing a one-day discharge protocol.

To the authors’ knowledge, there have only been a few investigations about parental satisfaction and discharge within 24 h of laparoscopic appendectomy for uncomplicated AA up to now. The outcomes of this study go along with previous findings showing high satisfaction after conducting good parental consultation for home management after hospital discharge within 24 h of surgery. The evaluation of the two-page questionnaire revealed that a greater majority of parents expressed the highest satisfaction for this treatment protocol already at the time of discharge. Only a minority reported a mediocre satisfaction. At the time of the outpatient appointment, the highest parental satisfaction increased to 97.2%. In the literature to this point, data collection has been performed using questionnaires or a follow-up telephone call [12,19,20,28]. The critical part of the success of this type of treatment protocol depends on good parent education and reassurance [12,20,28,31]. Ngo et al. reported that 81.6% of the parents were satisfied with early discharge and only 7% of parents would not choose an early discharge again [20]. Alkhoury et al. reported 87% of parents being satisfied postoperatively with the expeditious discharge. They observed a further increase up to 92% of parents when asked again in retrospect [12]. Yu et al. reported a high parental satisfaction of 88% [28]. Gee et al. reported in their study a slight increase in call consultations regarding pain control in the discharge group compared with their comparison group [19]. This study shows that the median of postoperative pain using the VAS was as high as 4 out of 10 on the first day and quickly declined to 0 on the fifth postoperative day with minimal necessity of pain medication.

Hospitalization has been related to several negative psychological outcomes for children, which include separation anxiety, tantrums, fear of strangers, eating problems, nightmares and enuresis. The shortening of the LOS may prevent children from experiencing these negative effects [13]. The median LOS in this study was 15 h. In the setting of this study, children were admitted to the pediatric ward after they had recovered in the postsurgical anesthetic care unit. Discharge was permitted when the child was drinking clear fluids and eating the first postoperative meal, without vomiting or nausea, and was able to walk to the bathroom with normal urination. Comparable studies showed a mean LOS after surgery of 5–8.8 h [12,18,31,34]. The LOS of this study was found to be twice as long as previously reported in the literature. The longer LOS may be attributed to the fact that some studies reported release from the ambulatory setting without ever admitting the patient to the pediatric surgical ward [12]. Moreover, patients in our study who had their appendectomy performed within earlier hours of the day showed a comparable postoperative LOS, while it was unlikely for patients presenting during evening hours to be released on the same day. This was previously described in the literature [18]. Nighttime discharges from hospitals are not feasible at this moment and in the Department where the study was conducted.

Acute appendicitis is a high-volume entity, and as such, effective resource allocation is warranted. Shortening the hospital stay can reduce costs for care providers. The exact cost for this institution is not possible to calculate, but Croatian Health Insurance Fund pays a sum of EUR 73.05 to the hospital for a trim day after appendectomy. Khan et al. published in 2013 a cost comparison study in which they meticulously listed the costs for surgical interventions and hospital stays. They were found to be comparable to those at our institution, giving an average cost for laparoscopic appendectomy of EUR 1.183.27 (OMR 479) and a cost for hospital stay per day of EUR 29.64 (OMR 12) [35]. In accordance with hospital policy, the policy of Department of Pediatric Surgery and a median LOS of 3 days [36], cost reduction per patient is estimated to be at least EUR 146.10 when using a one-day discharge protocol. When taking this into a one-year model for Department of Pediatric Surgery with an estimated 160 laparoscopic appendectomies for uncomplicated AA, a total reduction of approximately EUR 23.372.62 may be achieved. Yu et al. enrolled 185 patients into their discharge protocol and reported a reduction in costs for their facility of USD 351 per case and an estimated total cost reduction over the one-year period of the study of USD 64.584 [28]. Gee et al. found in their one-year study in 2016 the significantly lower median costs of stay for 382 discharged patients of USD 5.677 per patient, which can be calculated to a yearly median cost saving of more than USD 2 million [19]. Cheng et al. found in their study conducted over 28 months overall higher hospital charges for the 75 patients of the non-discharge group of up to USD 4000 per patient [34]. In Croatia, laparoscopic appendectomy is still mandatorily related with a hospitalization of more than 24 h. The results of this study, both in terms of medical safety and economic benefit, may be a cornerstone in reorganizing the standard of procedure for uncomplicated AA treated via laparoscopic appendectomy.

Finally, it is important to emphasize and repeat that the term “same day discharge” can be misleading, and maybe, for this type of discharge, a more correct term would be “discharge within 24 h after the surgery”.

This study shows success in implementing a same-day discharge protocol but shows several limitations. The results of a single-institution-based investigation are usually limited for general applicability. More clinical research and a multicenter type of study protocol are needed to better interpret the correlations among outcomes in larger cohorts. Nevertheless, the results may represent the general population, as the findings build on existing evidence that a one-day discharge protocol after laparoscopic appendectomy is safe in daily clinical practice. Moreover, the sample size shows sufficient patients to be generally applicable. Although the findings reflect the results of other studies, the lack of a comparison group harbors the risk of making erroneous conclusions about the outcomes of this study. Additionally, a seven-day postoperative follow-up period may be too short to establish the total amount of surgically related complications, although all immediate and early complications should be shown within the first six days of surgery. Lastly, the decision to use the 24 h treatment protocol implemented in this study was made by one of three surgeons intraoperatively. This bares the risk of increasing the selection bias. Other surgeons of the department were skeptical about the safety of this treatment protocol and decided not to be included for now. Nonetheless, inclusion was clearly defined with standardized intraoperative appendicitis grading, and there was no drop out observed in the patient group during follow-up, which strengthens the consistency of the results of this study.

## 5. Conclusions

This prospective cohort study showed that discharge within 24 h after laparoscopic appendectomy for uncomplicated AA in children is safe and feasible. Parental satisfaction is high with this treatment protocol. Via early discharge, hospitalization rates and potential psychological problems of children and parents are reduced. Additionally, reductions in costs for institutions are likely. With the proper algorithm and parent education, uncomplicated AA should be addressed this way.

## Figures and Tables

**Table 1:** Intraoperative appendicitis grading.

Uncomplicated Acute Appendicitis	
Grade 0	Macroscopically normal/histological endoappendicitis
Grade I	Inflamed appendix (hyperemia, edema ± fibrin)
**Complicated Acute Appendicitis**	
Grade II	Necrosis (a) Segmental (b) Involving the base
Grade III	Perforated–inflammatory tumor (a) With phlegmon (b) With <5 cm abscess (c) With >5 cm abscess
Grade IV	Perforated with diffuse peritonitis

**Table 2:** Demographic and clinical data of the patients.

Demographic Data	Value
Age (years); median (IQR)	11 (10, 14)
Gender; *n* (%)	
Male	115 (63.8)
Female	65 (46.2)
BMI (kg/m^2^) (IQR)	18.2 (15.5, 20.6)
Symptom duration (h); median (IQR)	24 (18, 32.5)
AIR score; median (IQR)	8 (7, 9)
White blood cells (×10^9^/L); median (IQR)	13.7 (11.6, 15.2)
Neutrophil count (%); median (IQR)	81.7 (79.9, 88.5)
C-reactive protein (mg/L); median (IQR)	21.1 (17.4; 32.5)
US total; *n* (%)	119 (100)
US positive; *n* (%)	90 (75.6)

IQR—interquartile range; BMI—body mass index; AIR—appendicitis inflammatory risk; US—ultrasound.

**Table 3:** Outcomes of treatment.

Outcome	Value
Duration of surgery (min); median (IQR)	20 (16, 25)
Length of stay (h); median (IQR)	15 (12, 19)
Appendiceal-base closure; *n* (%)	
Harmonic scalpel	155 (86.1)
Polymeric clip	25 (13.9)
Complications; *n* (%)	
Intraoperative	0
Postoperative	4 (2.2)
Readmissions; *n* (%)	4 (2.2)

IQR—interquartile range.

**Table 4:** Clavien–Dindo classification of postoperative complications.

Grade	*n* (%) (*n* = 4)	Total (*n* = 180)
I	0	0
II	4 (100)	4 (2.2)
III a	0	0
III b	0	0
IV a	0	0
IV b	0	0
V	0	0

**Table 5:** Parental satisfaction evaluation.

Parental Satisfaction	*n* (%)
Time of discharge	
Highest	155 (86.1)
Mediocre	25 (13.9)
Bad	0
Outpatient appointment	
Highest	175 (97.2)
Mediocre	5 (2.8)
Bad	0

## Data Availability

The data presented in this study are available upon request from the respective author. Due to the protection of personal data, the data are not publicly available.
